# Interplay of different synchronization modes and synaptic plasticity in a system of class I neurons

**DOI:** 10.1038/s41598-022-24001-2

**Published:** 2022-11-16

**Authors:** Irmantas Ratas, Kestutis Pyragas

**Affiliations:** Center for Physical Sciences and Technology, 10257 Vilnius, Lithuania

**Keywords:** Spike-timing-dependent plasticity, Dynamical systems, Statistical physics, thermodynamics and nonlinear dynamics

## Abstract

We analyze the effect of spike-timing-dependent plasticity (STDP) on a system of pulse-coupled class I neurons. Our research begins with a system of two mutually connected quadratic integrate-and-fire (QIF) neurons, which are canonical representatives of class I neurons. Along with various asymptotic modes previously observed in other neuronal models with plastic synapses, we found a stable synchronous mode characterized by unidirectional link from a slower neuron to a faster neuron. In this frequency-locked mode, the faster neuron emits multiple spikes per cycle of the slower neuron. We analytically obtain the Arnold tongues for this mode without STDP and with STDP. We also consider larger plastic networks of QIF neurons and show that the detected mode can manifest itself in such a way that slow neurons become pacemakers. As a result, slow and fast neurons can form large synchronous clusters that generate low-frequency oscillations. We demonstrate the generality of the results obtained with two connected QIF neurons using Wang–Buzsáki and Morris–Lecar biophysically plausible class I neuron models.

## Introduction

The study of brain rhythms and synchronization of oscillatory activity is currently one of the hottest topics in neuroscience. Under normal conditions, synchronization of oscillations is a mechanism for neural communication, which endows individual brain areas with the ability to perform specific tasks^1^. Conversely, extremely strong synchronization may impair brain function and cause various neurological disorders like Parkinson’s disease^2^, epilepsy^3,4^, and others^5^. Synchronization depends on the individual properties of neurons, as well as on the structure of the network and the strength of the coupling between neurons. In the case of weak coupling, the ability of individual neurons to synchronize is determined by their natural frequencies and phase response curves (PRC)^6,7^. Due to synaptic plasticity^8–10^, the structure of the neural network is not static. The weights of synaptic connections change depending on the activity of individual neurons. The activity of the neurons, in turn, depends on the connectivity. As a result, a feedback loop is established between neural dynamics and network structure.

In this paper, we investigate the dynamics of pulse-coupled class I neurons with synaptic weights governed by spike-timing-dependent plasticity^11,12^. Neuronal models with class I excitability generate action potentials with arbitrarily low frequency, depending on the strength of the applied current^13^. Since the frequency of neurons can vary greatly with current, such models are natural candidates for analyzing the effects of plasticity in neural systems with widely varying frequencies. In class I neurons, the transition from quiescence to spiking occurs via a saddle-node bifurcation on an invariant circle. The normal form equation near this bifurcation is known as the quadratic integrate-and-fire neuron model^14,15^. The QIF neuron is considered as the main model in our study, as it allows obtaining analytical results. In addition, we demonstrate the generality of our results using Wang–Buzsáki (WB)^16,17^ and Morris–Lecar (ML)^18,19^ biophysically plausible class I neuron models. Class I neurons generally have a purely positive PRC (also called type I PRC), indicating that perturbations always produce an advance (and not a delay) of their phase^20^. It has been shown that neurons with type I PRCs do not tend to synchronize in networks with fixed mutual excitatory synaptic connections, but can synchronize in directed acyclic networks^21^.

STDP is a phenomenon in which the precise timing of spikes affects the sign and magnitude of changes in synaptic strength. It is believed that STDP plays a crucial role in memory formation and maintenance^22^. Various forms of STDP have been observed in experiments^23–26^. We consider STDP with Hebbian learning rule, when presynaptic firing followed by a postsynaptic spike causes potentiation, and postsynaptic firing occurring before presynaptic firing leads to depression^8–10^. Effective modification of synaptic weight occurs only when the spike-timing difference is within a certain time interval, called the learning window. Typically, the learning windows for potentiation and depression are asymmetric^27–29^. In our study, we implement the classic additive pair-based STDP rule with asymmetric learning windows. STDP rules that depend on the neurons’ firing rates^30^ or other additional factors^31^ are beyond the scope of our consideration.

Over the past two decades, significant progress has been made in understanding the mechanism of STDP in the formation of connections in neural networks^32–38^ and the emergence of multistability in asymptotic network configurations^33,38–40^. From a theoretical point of view, the mechanism of connection formation is best understood by considering simplified models of two mutually coupled neurons with plastic synapses^33,38,40–42^. Models with pairwise interactions of neurons are more analytically tractable and provide important insights about the structures that STDP can produce in large networks^41^. Analysis of such models with STDP rules similar to those used in our article shows that in the case of asynchronous neuronal dynamics, plasticity tends to break neural connections^32,33,35,37,38^. In the case of synchronous dynamics, synapses in which high-frequency neurons are presynaptic tend to be potentiated, and connections from low-frequency neurons are weakened, so plasticity makes connections unidirectional from faster to slower neurons^32,33,35,37,38^. In this paper, we show that, unlike to results presented in previous works, STDP can produce opposite unidirectional connections from slower neurons to faster neurons. This can happen in frequency-locked mode, where the faster neuron emits multiple spikes per cycle of the slower neuron. Due to this effect, slow neurons in large plastic networks can become pacemakers. To our knowledge, no such effects of STDP have been reported in the literature.

## Model

We consider a system of *N* synaptically coupled class I neurons that generally can be presented by Hodgkin–Huxley-type equations of the form:

1a $$\begin{aligned} C_m\dot{v}_{i}= &\, F(v_i,{\textbf {w}}_i,\eta _i) + g \sum _{j=1} ^N W_{ij}(t) S_j(t), \end{aligned}$$

1b $$\begin{aligned} \dot{{\textbf {w}}}_i= & \, {\textbf {G}}(v_i,{\textbf {w}}_i, \gamma _i), \quad i=1,\ldots , N. \end{aligned}$$

Here $C_m$ is the membrane capacitance and $v_i$ is the membrane potential of the *i*th neuron. The function $F(v_i,{\textbf {w}}_i,\eta _i)$ describes the sum of currents flowing through the ion channels. Equation (1b) describes the dynamics of a recovery variable ${\textbf {w}}$ that generally is a vector variable, and the function ${\textbf {G}}(v_i,{\textbf {w}}_i, \gamma _i)$ represents the ionic channel dynamics. The parameters $\eta _i$ and $\gamma _i$ are constants that determine the heterogeneity of neurons. The functions *F* and ${\textbf {G}}$ are defined by a specific neuron model. The last term in the Eq. (1a) describes the interaction between neurons, where $g\ge 0$ is the homogeneous excitatory coupling. We assume that each neuron emits a spike when its membrane potential reaches the maximum. We approximate spikes by the Dirac delta function, so the spiking activity of the *j*th neuron can be written as

2 $$\begin{aligned} S_{j}(t)=\sum _{k|t_{j}^{(k)} \le t}\delta (t-t_{j}^{(k)}), \end{aligned}$$

where $t_{j}^{(k)}$ is the time of the *k*th spike of *j*th neuron. The synaptic weight $W_{ij}$ determines the strength of the connection between the presynaptic *j*-th neuron and the postsynaptic *i*-th neuron. We assume that the weights $W_{ij}$ (apart from the autaptic terms: $W_{ii}=0$) evolve in time according to a nearest-neighbor STDP rule. They change discretely in time and are updated at each spiking event. Assume that a specific *j*-th neuron fires at the time $t=t_j$. The change of the weights depends on time differences $\delta _{ji}=t_j-t_i^{(\texttt {last})}>0$, where $t_i^{(\texttt {last})}$ is the last firing time of the *i*th neuron. The weights $W_{ji}$ associated with directional links from the *i*th neurons to the *j*th neuron are potentiated, and the weights $W_{ij}$ associated with directional links from *j*th neuron to the *i*th neurons are depressed. Specifically, the STDP update rule is as follows:

3 $$\begin{aligned} W_{ji}(t_j)\leftarrow W_{ji}(t_j)+pe^{-\delta _{ji}/\tau _p}; \quad W_{ij}(t_j)\leftarrow W_{ij}(t_j)-de^{-\delta _{ji}/\tau _d}. \end{aligned}$$

Here $\tau _p$ ($\tau _d$) is the learning window over which post- (pre-) synaptic spikes induce synaptic potentiation (depression). Following experimental evidences^27–29^, we assume $\tau _p<\tau _d$. The parameters *p* and *d* represent the maximum update amplitudes for the potentiation and depression, respectively. We use an additive update rule with the requirement that the weights $W_{ij}$ do not go beyond the interval $0\le W_{ij}\le 1$. The maximum value of $W_{ij}$, equal to one, is chosen without loss of generality due to the factor *g* in Eq. (1a), which determines the global coupling strength. We assume that the parameters $p,d \ll 1$ are small, so that the synaptic weights change slowly compared to the characteristic time between firing events. Our main results concern the case $p=d$. For $p=d$ and $\tau _p < \tau _d$, there is a bias for synaptic depression, which facilitates competition between synapses.

We demonstrate our results with three different specific models of class I neurons, namely QIF neurons, WB neurons, and ML neurons. The simplest model of class I neurons is the QIF neuron. It does not contain the recovery variables and is obtained from Eq. (1a) by setting $C_m=1$ and $F(v_i,{\textbf {w}}_i,\eta _i)=v_i^2+\eta _i$^14^:

4 $$\begin{aligned} \dot{v}_i = v_i^2+\eta _i+g\sum _{j=1}^{N} W_{ij}(t) S_j(t). \end{aligned}$$

Recovery is implemented by an instantaneous reset of the membrane potential. Every moment when the membrane potential $v_i$ reaches the peak value $v_p$, the neuron emits a spike and its voltage is reset to the value $v_r$. Because of the quadratic nonlinearity, $v_i$ reaches infinity in a finite time, and this allows us to choose the threshold parameters as $v_p=-v_r=\infty$. With this assumption, the free ($g=0$) neuron with a positive parameter $\eta _i$ fires periodically with a period $T_i=\pi /\sqrt{\eta _i}$. The QIF neuron is the canonical model for the class I neurons near the spiking threshold^15^. By the change of variables

5 $$\begin{aligned} v_i= -\sqrt{\eta _i}\cot \left( \varphi _i/2 \right), \end{aligned}$$

the equation (4) can be transformed to the Winfree model^43,44^

6 $$\begin{aligned} \dot{\varphi }_i = \omega _i + g Z_i(\varphi _i) \sum _{j=1} ^N W_{ij}(t) S_j(t), \end{aligned}$$

where $\varphi _i \in (0, 2\pi ]$ is the phase, $\omega _i=2\pi /T_i$ is the natural frequency and

7 $$\begin{aligned} Z_i(\varphi )= 2\left[ 1-\cos (\varphi ) \right] / \omega _i \end{aligned}$$

is the PRC of the *i*th QIF neuron. In this representation, the *i*th neuron fires when its phase reaches $\varphi _i = 2\pi$. We emphasize that for spiking neurons ($\eta _i>0$) Eqs. (6) and (7) are equivalent to the original Eq. (4). Note that in the original Eq. (4), the coupling strength *g* can be arbitrarily large, hence the same is true for Eq. (6). The change in the phase of a given neuron at the moments of firing of other neurons can be calculated from the change in potential in the original model (4). In particular, the membrane potential of the *i*th neuron immediately after the *k*th spike of the *j*th neuron at time $t_j^{(k)}$ is updated as $v_i(t_j^{(k)}) \leftarrow v_i(t_j^{(k)})+gW_{ij}(t_j^{(k)})$, and the phase update is simply computed from the inverse transformation $\varphi _i=2 \hbox {arccot}(-v_i/\sqrt{\eta _i})$ of Eq. (5).

The hallmark of the Winfree model is its versatility. Any type of periodically spiking neurons described by the Eqs. (1a) and (1b) can be reduced to the Winfree model (6) provided that the coupling strength *g* is small. In this case, the PRC $Z_i(\varphi _i)$ depends on the specific form of Eq. (1a) and (1b) ﻿and can be obtained numerically from these equations. Thus, we also analyze the case of weakly coupled neurons WB and ML within the framework of the Winfree Eq. (6). The corresponding PRCs for WB and ML neurons are presented in “Biophysical neuron models”. Interaction between neurons is effective with weak coupling, if their natural frequencies satisfy the resonance conditions. Therefore, near resonances, the Winfree model makes it possible to obtain general analytical results.

## Results

In this section, we will first present the results of an analytical and numerical analysis of the asymptotic dynamics of two ($N=2$) mutually coupled class I neurons whose synaptic weights obey the STDP rule. Without loss of generality, we will assume that the first neuron is faster than the second, i.e. the natural frequencies (periods) of neurons satisfy the inequality $\omega _1>\omega _2$ ($T_1<T_2$). We then use these results to explain connectivity patterns emerging in larger ($N>2$) plastic networks.

Numerical simulation of two mutually coupled neurons QIF, WB or ML with synaptic weights regulated by the STDP rule shows that, depending on the parameters and initial conditions, their dynamics converge to three different asymptotic modes characterized by different sets of synaptic weights with different modes of synchronization.

**Mode (i):** $(W_{12 }, W_{21})\approx (1,0)$. Here at $t \rightarrow \infty$ the neurons become unidirectionally coupled so that the second (slower) neuron enslaves the first (faster) neuron with maximum synaptic weight $W_{12}$, and both oscillate at a slow frequency $\omega _2$ in synchronous mode characterized by the winding number $w=n/1$, where $n\ge 2$ is the number of spikes emitted at nonidentical intervals by the first neuron in one period of the second neuron.
**Mode (ii):** $(W_{12 }, W_{21})\approx (0,1)$. Here at $t \rightarrow \infty$ the opposite uniderectional coupling is established, i.e. the first (faster) neuron enslaves the second (slower) neuron with maximum synaptic weight $W_{21}$, and both oscillate at a fast frequency $\omega _1$ in synchronous mode with the winding number $w=1/1$ (the second neuron emits one spike in one period of the first neuron).
**Mode (iii):** $(W_{12 }, W_{21})\approx (0,0)$. Here at $t \rightarrow \infty$ both neurons becomes disconnected. Neurons are not synchronized and each of them oscillates at its own frequency.

A detailed analysis of these modes is especially convenient within the framework of the QIF neuron model. For this model, it was possible to analytically obtain frequency locking regions [Arnold tongues (AT)] at fixed synaptic weights and in the presence of STDP for any values of the coupling strength and frequency mismatch. We also managed to obtain general analytical expressions for the boundaries of AT for arbitrary class I neurons near resonances. The generality of the AT’s structure, as well as the reliability of analytical results near resonances, are confirmed by numerical simulation of biophysical models of WB and ML neurons. We then demonstrate how the asymptotic modes observed in two mutually coupled neurons manifest themselves in larger plastic networks. We consider networks consisting of two groups of slow and fast neurons with close natural frequencies within the groups, but significantly different frequencies between the groups. Depending on the initial conditions, such networks can evolve into various stable configurations. The peculiarity of these configurations is that slow neurons can enslave fast neurons and form clusters that oscillate synchronously at a low frequency. Below we describe these results in more detail.

**Figure 1:**
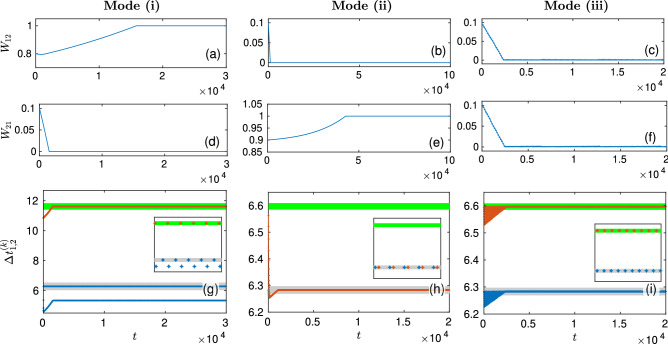
Convergence to asymptotic modes in two mutually coupled QIF neurons. Dynamics of (**a**–**f**) synaptic weights and (**g**–**i**) interspike intervals. The left column shows convergence to the synchronous asymptotic mode (i) with synaptic weights $(W_{12},W_{21}) \approx (1,0)$ and winding number $w=2/1$ (the first neuron fires two times in one period of the second neuron). The middle column shows convergence to the synchronous asymptotic mode (ii) with synaptic weights $(W_{12}, W_{21}) \approx (0,1)$ and winding number $w=1/1$ (the first neuron fires one time in one period of the second neuron). In the right column, the system converges to the asynchronous asymptotic mode (iii), where neurons becomes disconnected $(W_{12}, W_{21}) \approx (0,0)$. In panels (**g**–**i**), the interspike intervals of the first $\Delta t_1^{(k)}$ and second $\Delta t_2^{(k)}$ neurons are shown by blue and orange dots, respectively. The natural periods of the first and second neuron are presented by thick horizontal gray and green lines, respectively. The insets show the post-transient (asymptotic) dynamics of interspike intervals on an extended time scale. Parameters $T_1=2\pi$, $p=d=0.001$, $\tau _p=\pi /3$, $\tau _d = \pi$ are the same for all columns. For the left column $T_2 = 1.85 T_1$, $g=0.7$, and for the middle and right columns $T_2 = 1.05 T_1$, $g=0.15$.

Typical examples of the dynamics of two coupled QIF neurons demonstrating convergence to the asymptotic modes (i), (ii), and (iii) are shown in the left, middle, and right columns in Fig. 1, respectively. The top, middle and bottom rows show the dynamics of synaptic weights $W_{12}$, $W_{21}$ and interspike intervals $\Delta t_j^{(k)}=t^{(k)}_j-t ^{(k- 1)}_j$, respectively. The values of these variables were generated using the event-driven algorithm described in “Quadratic integrate-and-fire neurons” and displayed in the figure at discrete times $t=t^{(k)}_j$. In the left column, the ratio of the natural neuron periods is close to two, $T_2/T_1=1.85$. The system approaches the final asymptotic state in two stages. At the first stage, the synaptic weight $W_{21}$ approaches zero, and the unidirectional coupling from the slower neuron to the faster one leads to a frequency-locked mode with the winding number $w=2/1$. This can be seen from the dynamics of interspike intervals: the second neuron fires with its natural period $T_2$ (interspike intervals $\Delta t_2^{(k)}$ of the second neuron are shown by red dots), and the first neuron fires twice in the same period $T_2$ (interspike intervals $\Delta t_1^{(k)}$ of the first neuron are shown by blue dots). For a sufficiently large *k*, the blue dots are located on two separated horizontal lines, so the adjacent interspike intervals of the first neuron are not identical $\Delta t_1^{(k-1)} \ne \Delta t_1^{(k)}$, but the sum $\Delta t_1^{(k-1)} + \Delta t_1^{(k)}$ is close to the interspike interval $\Delta t_2^{(k)}$ of the second neuron. This means that the second neuron enslaves the first neuron and makes it oscillate with the natural period $T_2$ of the slow neuron, emitting two spikes during this period at irregular intervals. At the second stage, the synaptic weight $W_{12}$ reaches its maximum value equal to one. In the middle column, the ratio of the natural neuron periods is close to one, $T_2/T_1=1.05$. Again, the system approaches the final asymptotic state in two stages. At the first stage, the synaptic weight $W_{12}$ approaches zero, and the unidirectional coupling from the slower neuron to the faster one leads to a frequency-locked mode with the winding number $w=1/1$. Now both neurons fire with the period $T_1$ of the faster neuron, and the slower neuron emits one spike during this period. At the second stage, the synaptic weight $W_{21}$ reaches its maximum value equal to one. In the right column, the parameter values are the same as in the middle column, but the initial values of the synaptic weights $W_{12}$ and $W_{21}$ are different. Here, both synaptic weights approach zero almost simultaneously, and each of them fires independently with its own natural period.

**Figure 2:**
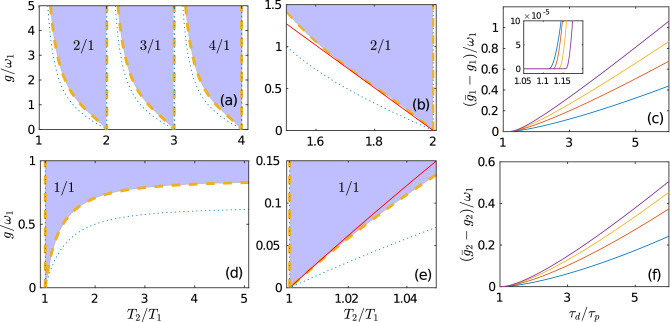
Effect of STDP on the synchronization of two QIF neurons. The top and bottom rows correspond to modes (i) and (ii), respectively. Arnold tongues of (**a**) synchronous asymptotic mode (i) with indicating winding numbers $w=n/1$ ($n=2,3,4$ are the number of spikes generated by the first neuron in one period of the second neuron) and (**d**) synchronous asymptotic mode (ii) with the winding number $w=1/1$. Blue dotted and yellow dashed curves show the boundaries of ATs for fixed synaptic weights and in the presence of STDP, respectively. Analytic expressions for these boundaries are presented in “Quadratic integrate-and-fire neurons”. The colored areas mark different frequency locking zones obtained by direct numerical simulation of two coupled QIF neurons in the presence of STDP. (**b**,**e**) Enlarged areas of (**a**,**d**). Here, additional red lines show approximations of AT boundaries near resonances, defined by the general analytical expressions (35) and (38), which are valid for any class I neurons. (**c**,**f**) Difference of AT boundaries with STDP ($\bar{g_i}$) and without STDP ($g_i$) depending on the ratio of STDP learning windows $\tau _d/\tau _p$. Curves of different colors correspond to different values of $T_2/T_1$. In panel (**c**), $T_2/T_1$: 1.9—purple, 1.8—yellow, 1.7—red, and 1.6—blue. In panel (**f**), $T_2/T_1$: 1.2—purple, 1.15—yellow, 1.1—red, 1.05—blue. Values of other parameters: $T_1=2\pi$, $p=d=0.001$, $\tau _p=\pi /3$, $\tau _d = \pi$. The inset in (**c**) shows the enlarged area around $\tau _d/\tau _p \approx 1$.

The existence of synchronous asymptotic modes (i) and (ii) depends on both the neuron parameters and the STDP parameters. In “STDP stability conditions for unidirectionally coupled neurons”, we derived general STDP stability conditions for these modes. Analytic expressions for modes (i) and (ii) are given by Eqs. (9) and (11), respectively. These conditions facilitate the search for frequency locking regions in the parameter plane $(T_2/T_1, g)$, called Arnold tongues. For QIF neurons, this problem admits a purely analytical solution, which is described in detail in “Quadratic integrate-and-fire neurons”. First, we found the coupling strength $g_1$, which defines the boundaries of the ATs with fixed synaptic weights $(W_{12},W_{21}) = (1,0)$ and the coupling strength $g_2$, which defines ATs with fixed synaptic weights $(W_{12},W_{21}) = (0,1)$. They are defined by the Eqs. (14) and (18), respectively, and are shown in Fig. 2 as blue dotted curves. Then we found out how these boundaries change when the STDP stability conditions are taken into account. The boundaries of ATs $\bar{g}_1$ for mode (i) and $\bar{g}_2$ for mode (ii) in the presence of the STDP are defined by Eqs. (21) and (23), respectively, and are shown by the yellow dashed curves. These curves coincide with the boundaries of the colored areas in Fig. 2, which represent synchronization domains (ATs) obtained by direct numerical simulation of the dynamics of two QIF neurons coupled by plastic synapses. The resonant structure of ATs is preserved in the presence of weak noise, see details in the Supplementary Information.

In general, ATs in the presence of STDP are inside ATs with fixed synaptic weights, $\bar{g}_{1,2} \ge g_{1,2}$. Figure 2c,f show the differences $\bar{g}_{1}-g_1$ and $\bar{g}_{2}-g_2$ depending on the ratio of the STDP learning windows $\tau _d/\tau _p$ for different values of $T_2/T_1$. These differences increase as $\tau _d/\tau _p$ increases and the ratio of natural periods of neurons $T_2/T_1$ moves away from resonance. Interestingly, for $\tau _d$ close to $\tau _p$ the ATs with and without STDP may coincide. This can be seen in the inset to Fig. 2c: the difference $\bar{g}_{1}-g_1$ vanishes on a finite interval of $\tau _d/\tau _p$ axis near unity.

The above analytical results give an accurate description of ATs for QIF neurons in the entire parameter plane $(T_2/T_1, g)$. We have generalized these results to arbitrary class I neurons for $T_2/T_1$ values close to resonances, when $T_2/T_1 \approx n$, $n=1,2,3,\ldots$ (see “General results near resonances” for details). Near resonances, synchronization occurs at a low coupling strength *g* (see Fig. 2), and this allows us to reduce any neuron model to the Winfree Eq. (6). Then the synchronization properties of a particular neuron model are determined by its PRC. We have shown that the ATs of two pulse-coupled class I neurons depend only on the local characteristics of the PRC near its absolute maximum. To obtain explicit analytical results, we approximated this maximum with a parabola. Equations (35) and (38) determine the approximate boundaries of ATs near resonances for modes (i) and (ii), respectively. They apply to any class I pulse-coupled neurons with plastic synapses. Figure 2b,e confirm the validity of these equations for the exactly solvable QIF neuron model near resonances $T_2/T_1 = 2$ and $T_2/T_1 =1$, respectively. The red lines approximating the boundaries of ATs by the formulas  (35) and (38) are in good agreement with the exact results shown by the yellow dashed curves.

**Figure 3:**
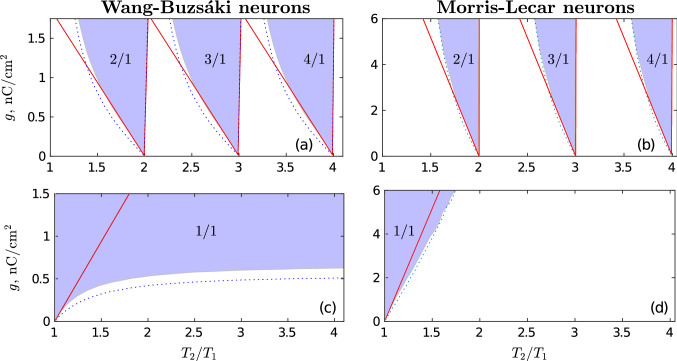
Effect of STDP on the synchronization of WB and ML neurons. The left column corresponds to two coupled Wang–Buzsáki neurons, and the right column represents two coupled Morris–Lecar neurons. The top and bottom rows correspond to modes (i) and (ii), respectively. (**a**,**b**) Arnold tongues of synchronous asymptotic mode (i) with different winding numbers $w=n/1$, $n=2,3$, and 4. (**c**,**d**) Arnold tongues of synchronous asymptotic mode (ii) with the winding number $w=1/1$. As in Fig. 2, the blue dotted curves show the boundaries of ATs for fixed synaptic weights. The colored areas mark different frequency locking zones obtained by direct numerical simulation of two coupled neurons in the presence of STDP. The red lines show approximations of AT boundaries near resonances, defined by the general analytical expressions (35) and (38). The equations of the WB and ML neuron models and their parameters are given in “Biophysical neuron models”. Values of other parameters: (**a**,**c**) $T_1= 500$, $p=d=0.002$, $\tau _d = T_1/2$, $\tau _p=\tau _d/3$; (**b**,**d**) $T_1 = 86.27$, $p=d=0.002$, $\tau _d = T_1/2$, $\tau _p = \tau _d/4$.

To verify the generality of the results obtained with the QIF neurons, we performed a similar analysis for more complex WB and ML biophysical neuron models. The results are presented in Fig. 3. Here, as in Fig. 2, the blue dotted curves are the boundaries of ATs with fixed synaptic weights, colored areas show numerically estimated synchronization domains (ATs) of two neurons coupled by plastic synapses and red lines are analytical approximations of the boundaries of ATs in the presence of STDP. The red lines are in good agreement with the boundaries of colored areas in the vicinity of resonances. This confirms the validity of general approximate analytical formulas (35) and (38) for WB and ML neurons. Comparing the results presented in Figs. 2a,d and 3 we see that the structure of ATs is the same for all three types of neurons: QIF, WB, and ML. Thus, the simple QIF neuron model qualitatively correctly describes the interaction of different synchronization modes and STDP for class I neurons and may serve as a basic model for analyzing the formation of connectivity patterns in large plastic networks consisting of class I neurons.

**Figure 4:**
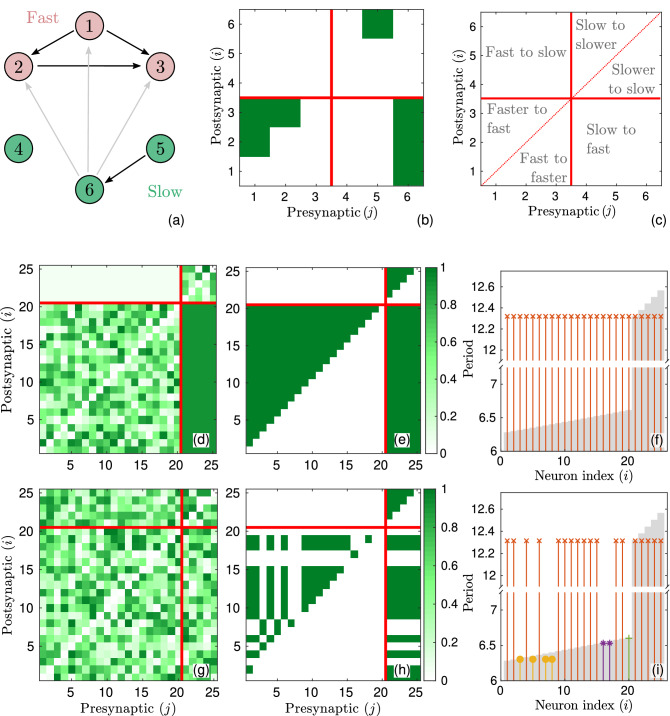
Examples of connectivity patterns emerging in plastic networks of QIF neurons. (**a**–**c**) An example of a network of six neurons, the first three of which are fast and the next three are slow. (**a**) One of the possible network configurations obtained in the post-transient regime with specific initial conditions. The arrows indicate the direction of the connections. All connections are realized with the maximum synaptic weight. (**b**) Asymptotic values of the elements of the matrix $W_{ij}$ shown in color. This matrix corresponds to the network configuration depicted in panel (**a**). Panel (**c**) explains the structure of connections in the matrix $W_{ij}$. Red vertical and horizontal lines separate the regions of slow and fast neurons. (**d**–**i**) Two examples of the initial and asymptotic states of a network consisting of 20 fast and 5 slow neurons. (**d**) Initial matrix $W_{ij}$ with partially random and partially deterministic choice of elements (see main text for details). (**e**) The asymptotic values of the matrix $W_{ij}$ obtained from the initial matrix shown in panel (**d**). Panel (**f**) shows the distribution of natural (gray bars) and actual (vertical red lines ending in crosses) periods of neurons in the post-transient regime. (**g**) The initial matrix $W_{ij}$, all elements of which are chosen randomly from the interval [0, 1]. (**h**,**i**) Same as (**e**,**f**) but for the initial matrix shown in (**g**). The coupling strength is assumed to be $g=0.25$. The STDP parameters are the same as in the case of two neurons.

Examples of connectivity patterns emerging in the plastic networks of QIF neurons are shown in Fig. 4. We consider heterogeneous networks consisting of two groups of neurons, fast and slow. The natural periods of neurons in each group differ slightly, while the natural periods between groups differ by about a factor of two. The neurons are numbered in such a way that their natural periods form an ascending sequence $T_1<T_2< \cdots < T_N$. Depending on the initial values of the synaptic weights $W_{ij}$, the network can develop into many different asymptotic configurations. All these configurations can be explained in terms of three possible asymptotic modes observed in a system of two coupled neurons. First consider a simple example of a network consisting of three fast neurons ($i=1,2,3$) with natural periods close to $2\pi$ and three slow neurons ($i=4,5,6$) with natural periods close to $4\pi$. One of the asymptotic configurations that can occur in this network is shown in the top row of Fig. 4. Panel (a) explicitly shows the connections between neurons and panel (b) represents the asymptotic values of the matrix $W_{ij}$ elements corresponding to this configuration, depicted in colors. Panel (c) explains the structure of connections in the matrix $W_{ij}$. On the panel (a), we see that one of the neurons ($i = 4$) is disconnected from other neurons of the network. This fact can be explained as a manifestation of mode (iii) observed in the system of two coupled neurons. The coupling between remaining neurons ($i\ne 4$) is unidirectional. This fact can be interpreted as a manifestation of modes (i) and (ii). The directions of connection within the groups of slow and fast neurons are indicated by black arrows, and between the groups of slow and fast neurons are indicated by gray arrows. In the group of slow neurons, the faster 5th neuron enslaves the slower 6th neuron [manifestation of mode (ii)]. Similarly, in the group of fast neurons, the fastest 1st neuron enslaves the slower 2nd and 3rd neurons, and the faster 2nd neuron enslaves the slower 3rd neuron. The most interesting property of this configuration is associated with mode (i): the slow 6th neuron enslaves the entire group of fast neurons, so all neurons in the network, except for the 4th, oscillate with a common period equal to the natural period of the slow 5th neuron.

Connectivity patterns emerging in larger plastic networks can be explained in a similar way. The middle and bottom rows in Fig. 4 show two examples of the initial and asymptotic states of the network consisting of $N=25$ QIF neurons. In both examples, the network parameters are the same, only the initial values of the matrix $W_{ij}$ differ. The first 20 neurons of the network are fast with natural periods equidistantly distributed in the interval [6.28, 6.61], and the last 5 neurons are slow with natural periods in the interval [12.31, 12.56]. The distribution of natural periods of neurons is shown by gray bars on panels (f) and (i). For the first example, the initial and final states of the matrix $W_{ij}$ are shown on panels (d) and (e), respectively. The initial synaptic weights $W_{ij}$ within the group of fast neurons and within the group of slow neurons are chosen randomly from the interval [0, 1], and the initial synaptic weights from fast to slow and from slow to fast neurons are taken to be 0.05 and 0.9 respectively. These initial conditions lead to the network configuration represented by the matrix $W_{ij}$ shown on panel (e). In the group of fast neurons, each neuron is unidirectionally connected to all faster neurons. The same thing happens in the group of slow neurons. Again, the manifestation of mode (i) gives the most interesting results. Each slow neuron is unidirectionally connected to all fast neurons, and all neurons of the network oscillate with a common period equal to the natural period of the 21st neuron, the fastest in the group of slow neurons. Actual periods of neurons in post-transient regime are shown in panel (e) by vertical red lines ending in crosses. Thus, the fastest neuron in the group of slow neurons becomes the pacemaker and enslaves the entire network.

More complex connectivity patterns can emerge when all initial elements of the matrix $W_{ij}$ are chosen randomly. Such an example is presented in the bottom row of Fig. 4. Now the network splits up into several isolated clusters. The 3rd, 5th, 7th and 8th neurons form a cluster of fast neurons oscillating with the natural period of the 3rd neuron, the fastest in this cluster. On the panel (i), the neurons of this cluster are marked with fat yellow dots. The 16th and 17th neurons, marked with blue asterisks, form another cluster of fast neurons, oscillating with the natural period of the 16th neuron. The above clusters arise from mode (i). The 20th neuron in a group of fast neurons is disconnected from all other neurons. This is a manifestation of mode (iii). All the remaining neurons marked with red crosses form a cluster of slowly oscillating neurons. This cluster is the result of the manifestation of modes (i) and (ii). The fastest 21st neuron in the group of slow neurons becomes the pacemaker and enslaves all the neurons of this cluster. Although in the above examples the ratio of natural periods in the groups of slow and fast neurons was chosen close to 2, we note that a slow neuron can become a pacemaker even in a “non-resonant” network when the ratio of natural periods in the groups differs significantly from 2. However, in this case, larger values of the coupling strength *g* are required. The effect of turning a slow neuron into a pacemaker is also resistant to external noise. See the Supplementary Information for more details.

## Discussion

We analyzed the dynamics of a system of class I pulse-coupled neurons in the presence of STDP. Class I neurons are characterized by a positive PRC, so any external perturbations affecting such neurons can only shift their phases forward. This property plays an important role in the formation of various synchronization modes in the system of interacting neurons. The synaptic weights controlled by STDP affect the synchronization mode. The synchronization mode, in turn, affects the performance of STDP. The interplay of the synchronization mode and STDP determines the asymptotic dynamics of the system. In our study, we mainly focused on the analysis of the effects of STDP in the model of two connected QIF neurons, which are the canonical representatives of class I neurons. Such a relatively simple model made it possible to obtain the main results in an analytical form. Three different asymptotic modes have been found in this system, denoted as (i), (ii), and (iii). The first two modes are synchronous and are characterized by unidirectional connections between neurons, while mode (iii) is asynchronous with broken connections. The effect of breaking connections in the presence of STDP has been previously observed in many different models^33,38,40,41^. The synchronous mode (ii) characterized by a unidirectional connection from a faster to a slower neuron has also been reported in many publications^32,33,35,37,38^. However, the synchronous mode (i) with a unidirectional connection from a slower neuron to a faster one, as far as we know, has not been observed.

The synchronous mode (i) is characterized by a winding number greater than one, which means that the faster neuron emits several spikes in one cycle of the slower neuron. This mode has a resonant structure of ATs on the parameter plain $(T_2/T_1,g)$, where $T_2/T_1$ is the ratio of the natural period of the slower neuron to the natural period of the faster neuron, and *g* is the coupling strength. For QIF neurons, the boundaries of ATs were obtained analytically both at fixed synaptic weights and in the presence of STDP. Near resonances $T_2/T_1 \approx n$, where *n* is a natural number, we generalized these analytical results for an arbitrary class I neuron model and confirmed their correctness on WB^16,17^ and ML ^18,19^ biophysically plausible class I neuron models.

The asymptotic modes observed in a system of two connected neurons allowed us to explain the connectivity patterns emerging in larger networks of class I neurons connected by plastic synapses. We considered a network consisting of two groups of neurons with significantly different natural frequencies between groups and similar frequencies within groups. Our analysis showed that, depending on the initial conditions, the network can evolve into many different stable configurations. The most interesting feature of these configurations is that slow neurons can become pacemakers in the network. This is explained as a manifestation of the synchronous mode (i) observed in the system of two connected neurons. In the network, STDP forms unidirectional links from slow to fast neurons, forcing the fast neurons to oscillate at a low frequency. As a result, slow and fast neurons can form large synchronous clusters generating low-frequency oscillations.

Understanding the mechanisms of connectivity formation in plastic neural networks is important when developing various stimulation techniques for long-lasting network desynchronization^39,45–48^. These techniques exploit plasticity-mediated multistability and aim to move the neural network from stable states characterized by abnormally strong synchrony and correspondingly increased synaptic weights to stable states with reduced synchrony and reduced synaptic weights. The reconfigured network structure induced by stimulation provides a long-lasting desynchronization that persists after the termination of stimulation. Long-lasting desynchronization techniques are desirable therapeutic tools for the treatment of Parkinson’s disease^49–51^. We hope that the model of the plastic network of QIF neurons, extended by various stimulation protocols, will become a suitable object for testing and developing various long-lasting desynchronization techniques. The event-driven algorithm provides efficient numerical simulation of such a model.

It should be noted that aberrant hypersynchronized states of certain areas of the brain do not occur in all brain disorders. Particularly, in Alzheimer’s disease, cumulative evidence indicates that desynchronization of neuronal firing might underlie the disease progression, and providing re-synchronization of neuronal networks is on the focus of the current therapeutical approaches^52–56^.

## Methods

### STDP stability conditions for unidirectionally coupled neurons

By numerical simulation of two mutually coupled class I neurons with plastic synapses, we found three asymptotic modes, denoted as (i), (ii), and (iii). Examples of convergence to these modes for QIF neurons are shown in Fig. 1. Based on these results, we derive general stability conditions for unidirectionally coupled neurons in synchronous modes (i) and (ii).

#### Mode (i)

**Figure 5:**
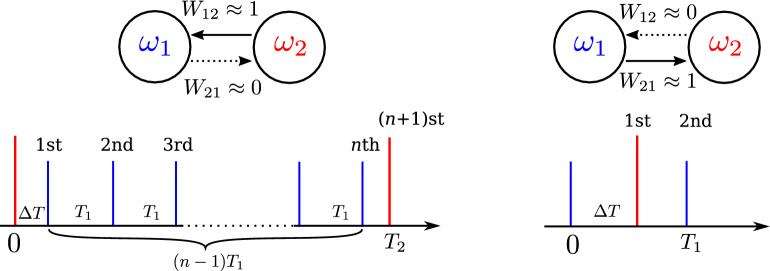
Connection diagrams and sequences of events for asymptotic modes (i) and (ii). The left and right columns correspond to modes (i) and (ii), respectively. The top row shows connection diagrams. Solid (dotted) arrows denote connections with maximum (vanishing) synaptic weight. The bottom row shows the sequences of events for the frequency-locked mode with the winding number $w=n/1$ (left) and $w=1/1$ (right). The short blue and long red vertical lines show the firing times of the first and second neurons, respectively. Event numbers are indicated above the lines.

The schematic diagram of this mode is shown in the left column of Fig. 5. Here, the coupling is unidirectional from the second (slow) to the first (fast) neuron with $(W_{12},W_{21}) \approx (1,0)$. The second neuron is practically independent of the first one and fires with its natural period $T_2$. In the frequency-locked mode with the winding number $w=n/1$ the first neuron fires *n* times in one period of the second neuron. We denote the steady-state delay time between the first firing of the first neuron and first firing of the second neuron in the synchronization cycle as $\Delta T$. For a particular neuron model, the value of $\Delta T$ is easy to obtain numerically. Later we will express $\Delta T$ in terms of the phase of the neuron. Let us estimate the changes in synaptic weights for $(n+1)$ events occurring in one synchronization cycle. The first event is related to the firing of the first neuron at $t=\Delta T$. Due to the STDP rule, the weight of $W_{12}$ will increase by $\Delta W_{12} = p \exp (- \Delta T/\tau _p)$, and the weight of $W_{21}$ will decrease by $\Delta W_{21} = -d \exp (- \Delta T/\tau _d)$. Similarly, for each firing of the first neuron (events with numbers $j<n+1$), the weight of $W_{12}$ will increase by $\Delta W_{12} = p \exp \left\{ - [ (j-1)T_1+\Delta T]/\tau _p \right\}$ and the weight of $W_{21}$ will decrease by $\Delta W_{21} = -d \exp \left\{ - [(j-1)T_1+\Delta T]/\tau _d \right\}$. The synchronization cycle ends with the $(n+1)$st event, at which the second neuron fires. Here, the weight of $W_{12}$ will decrease by $\Delta W_{12} = -d \exp \left\{ - [T_2-(n-1)T_1-\Delta T]/\tau _d \right\}$ and the weight of $W_{21}$ will increase by $\Delta W_{21} = p \exp \left\{ - [T_2- (n-1)T_1-\Delta T]/\tau _p \right\}$. Summing up all the changes of the synaptic weights during the synchronization cycle, we get

8a $$\begin{aligned} \Delta \bar{W}_{12}= &\, p \sum _{j=0}^{n-1} \exp \left( -\frac{j T_1+\Delta T}{\tau _p} \right) - d \exp \left( - \frac{T_2-(n-1)T_1-\Delta T}{\tau _d} \right) , \end{aligned}$$

8b $$\begin{aligned} \Delta \bar{W}_{21}= &\, p \exp \left( - \frac{T_2-(n-1)T_1-\Delta T}{\tau _p} \right) - d \sum _{j=0}^{n-1} \exp \left( -\frac{j T_1+\Delta T}{\tau _d} \right) . \end{aligned}$$

Because of the small parameters *p* and *d*, changes in synaptic weights are small, so that during the synchronization cycle $W_{12}$ remains close to zero and $W_{21}$ remains close to one. Recall that we use additive (hard) boundary conditions, i.e. when the synaptic weights $W_{ij}$ go outside the interval [0, 1], they are numerically “forced” to return to this interval. Then STDP provides stable unidirectional coupling if $W_{12}$ increases and $W_{21}$ decreases during one synchronization cycle without taking into account the boundary conditions, i.e. the STDP stability conditions are as follows: $\Delta \bar{ W}_{12}>0$ and $\Delta \bar{W}_{21}<0$. When the rates of potentiation and depression are the same, $p=d$, and the synaptic time windows satisfy the inequality $\tau _p\leqslant \tau _d$, these conditions can be simplified to

9 $$\begin{aligned} \frac{\Delta T}{T_1} < Q_1 \equiv \frac{1}{1+\tau _d/\tau _p} \left\{ \frac{T_2}{T_1}-n+1+\frac{\tau _d}{T_1} \ln \left[ \sum _{j=0}^{n-1} \exp \left( -\frac{jT_1}{\tau _p} \right) \right] \right\} . \end{aligned}$$

#### Mode (ii)

The schematic diagram of this mode is shown in the right column of Fig. 5. Here, the coupling is unidirectional from the first (fast) to the second (slow) neuron with $(W_{12},W_{21}) \approx (0,1)$. In this mode, the winding number is $w=1/1$, which means that the second neuron fires once during one period of the first neuron. The changes of synaptic weights during this period are:

10a $$\begin{aligned} \Delta \bar{W}_{12}= &\, p \exp \left( -\frac{T_1-\Delta T}{\tau _p} \right) -d \exp \left( -\frac{\Delta T}{\tau _d} \right) , \end{aligned}$$

10b $$\begin{aligned} \Delta \bar{W}_{21}= & \, p \exp \left( -\frac{\Delta T}{\tau _p} \right) -d \exp \left( -\frac{T_1-\Delta T}{\tau _d} \right) . \end{aligned}$$

STDP provides stable unidirectional coupling if, without taking into account the boundary conditions, $\Delta \bar{W}_{12}<0$ and $\Delta \bar{W}_{21}>0$. For $p=d$, and $\tau _p\leqslant \tau _d$, these conditions can be simplified to

11 $$\begin{aligned} \frac{\Delta T}{T_2} < Q_2 \equiv \frac{1}{1+\tau _d/\tau _p}\frac{T_1}{T_2}. \end{aligned}$$

### Quadratic integrate-and-fire neurons

#### Event-driven algorithm

A plastic QIF neural network, described by Eqs. (4) or (6), can be simulated by an event-driven algorithm. Here we describe this algorithm in terms of the Winfree model using Eqs. (6) with the PRC (7). The dynamic variables of the network are phases $\varphi _i(t)$ and synaptic weights $W_{ij}(t)$ for $i,j=1,\ldots , N$. Between firing events, neurons do not interact, and their phases increase linearly. The firing of any neuron leads to a discrete change in phases and synaptic weights. Assume that at time *t* the values of the dynamic variables of the network are known. Then the event-driven algorithm looks like this:

1. For each neuron, calculate the expected time interval $\Delta t_i=[2\pi -\varphi _i(t)]/\omega _i$ until its next firing.
2. Find the minimal time $\Delta t_j=\min _i(\Delta t_i) \equiv \Delta t$ and the index *j* of the neuron that should fire first.
3. Compute the phases $\varphi _i(t+\Delta t_j)=2 \hbox {arccot}\left[ \cot \left( \frac{\varphi _i(t)+\omega _i \Delta t_j}{2} \right) - \frac{2g}{\omega } W_{ij}(t) \right]$ of all $i\ne j$ neurons immediately after firing of the *j*th neuron.
4. Update synaptic weights $W_{ij}$ according to the STDP rule Eq. (3).
5. Update time $t \leftarrow t+\Delta t_j$ and repeat steps 1–5.

#### Arnold tongues with fixed synaptic weights

First, consider a system of two ($N=2$) unidirectionally coupled QIF neurons with fixed synaptic weights $(W_{12}, W_{21})=(1,0)$, disregarding synaptic plasticity. In this case, the second neuron is free and fires with a natural period $T_2$ that is larger than the natural period $T_1$ of the first neuron. At the instants of firing of the second neuron, the phase $\varphi _1$ of the first neuron satisfies the mapping

12 $$\begin{aligned} \varphi _1^{(k+1)} = 2 \hbox {arccot}\left[ \cot \left( \frac{\varphi _1^{(k)}+\omega _1 T_2}{2} \right) - \frac{2g}{\omega _1} \right] . \end{aligned}$$

Here $\varphi _1^{(k)}$ and $\varphi _1^{(k+1)}$ are the phases of the first neuron immediately after the *k*th and $(k+1)$st spikes of the second neuron, respectively. The fixed points of the map are determined by the equation $\varphi ^{(k+1)} =\varphi ^{(k)}\equiv \bar{\varphi }_1$. A stable solution to this equation gives the stationary phase of the first neuron when it is synchronized with the second neuron,

13 $$\begin{aligned} \bar{\varphi }_1=\pi +2\arctan \left( G+\sqrt{G^{2}-1-2G\cot \left( \pi T_2/T_1\right) }\right) , \end{aligned}$$

where $G=g/\omega _1$. Synchronization occurs if the root in this equation is real. Equating the expression under the root to zero, $G^{2}-1-2G\cot \left( \pi T_2/T_1\right) =0$, we find the critical (minimal) value $g_1=G \omega _1$ of the coupling strength,

14 $$\begin{aligned} g_{1} = \omega _1 \cot \left[ \frac{\pi }{2}\left( \frac{T_2}{T_1}-n+1\right) \right] , \quad n-1<\frac{T_2}{T_1} \le n, \end{aligned}$$

at which synchronization occurs. The dependence of $g_1$ on $T_2/T_1$ defines the boundaries of Arnold tongues. The stationary phase on these boundaries is

15 $$\begin{aligned} \bar{\varphi }_1^A=2\pi \left[ 1-\frac{1}{2} \left( \frac{T_2}{T_1}-n+1\right) \right] , \quad n-1<\frac{T_2}{T_1} \le n. \end{aligned}$$

Here and in Eq. (14) $n=2,3,\ldots ,\infty$ are natural numbers that define the Arnold tongues with the winding numbers $w=n/1$. In terms of phase, the winding number determines the number of revolutions made by the phase of the response neuron when the phase of the driving neuron makes one revolution, i.e. varies from 0 to $2\pi$. The boundaries of ATs determined by the Eq. (14) are shown by blue dotted curves in Fig. 2a,b.

We now consider unidirectional coupling with synaptic weights $(W_{12}, W_{21})=(0,1)$, when a free fast neuron excites a slower neuron. At the instants of firing of the first neuron, the phase $\varphi _2$ of the second neuron satisfies the mapping

16 $$\begin{aligned} \varphi _2^{(k+1)} = 2 \hbox {arccot}\left[ \cot \left( \frac{\varphi _2^{(k)}+\omega _2 T_1}{2} \right) - \frac{2g}{\omega _2} \right] . \end{aligned}$$

A stable fixed point $\varphi _2^{(k+1)} =\varphi _2^{(k)}\equiv \bar{\varphi }_2$ of this map gives the stationary phase of the second neuron when it is synchronized with the first neuron,

17 $$\begin{aligned} \bar{\varphi }_2=\pi +2\arctan \left( G+\sqrt{G^{2}-1-2G\cot \left( \pi T_1/T_2\right) }\right) , \end{aligned}$$

were $G=g/\omega _2$. Equating the expression under the root to zero, $G^{2}-1-2G\cot \left( \pi T_1/T_2\right) =0$, we find the boundary $g_2=G\omega _2$ of AT with the winding number $w=1/1$:

18 $$\begin{aligned} g_{2} = \omega _2 \cot \left( \frac{\pi }{2}\frac{T_1}{T_2}\right) \end{aligned}$$

The stationary phase on this boundary is

19 $$\begin{aligned} \bar{\varphi }_2^A=2\pi \left( 1-\frac{1}{2} \frac{T_1}{T_2}\right) . \end{aligned}$$

The boundary of AT, defined by Eq. (18), is shown by blue dotted curves in Fig. 2c,d.

#### Arnold tongues in the presence of STDP

We can now estimate how Arnold tongues change when STDP is taken into account. Unidirectional coupling $(W_{12},W_{21})=(1,0)$ is stable in the presence of STDP if the inequality (9) is satisfied. In this inequality, the time $\Delta T$ can be expressed in terms of the stationary phase $\bar{\varphi }_1$ as $\Delta T=(2\pi -\bar{\varphi }_1)/\omega _1$. Here we have used the fact that the QIF neuron fires when its phase reaches $2\pi$. As a result, the STDP stability condition (9) can be written as

20 $$\begin{aligned} \bar{\varphi }_1 >2\pi (1 -Q_1). \end{aligned}$$

First let us check whether the stability condition (20) is met on the boundaries of ATs Eq. (14) obtained with fixed synaptic weights. Substituting $\bar{\varphi }_1=\bar{\varphi }_1^A$ into inequality (20), we have $\bar{\varphi }_1^A >2\pi (1 -Q_1)$ or $Q_1>(T_2/T_1-n+1)/2$. If this inequality is met, then the boundaries $\bar{g}_1$ of ATs in the presence of STDP will coincide with the boundaries $g_1$ of ATs obtained with fixed synaptic weights. If the above inequality is not met then boundaries $\bar{g}_1$ are estimated by replacing the inequality (20) with equality and substituting into it the stationary phase $\bar{\varphi }_1$ from the Eq. (13). As a result, we get the following expression for the boundaries of ATs with the winding numbers $w=n/1$ in the presence of STDP:

21 $$\begin{aligned} \bar{g}_1 = {\left\{ \begin{array}{ll} g_1 & \text {if } Q_1 \ge (T_2/T_1-n+1)/2, \\ \omega _1 \left\{ 2 \sin ^2(\pi Q_1) \left[ \cot (\pi Q_1)-\cot (\pi T_2/T_1) \right] \right\} ^{-1} &{} \text {if } Q_1<(T_2/T_1-n+1)/2. \end{array}\right. } \end{aligned}$$

They are shown by yellow dashed curves in Fig. 2a,b. In Fig. 2c, we show the difference $\bar{g}_1-g_1$ between the boundaries of ATs in the presence of STDP and without STDP as functions of a ratio $\tau _d/\tau _p$ for different values of $T_2/T_1$. On the inset, the area where $\tau _d \approx \tau _p$ is enlarged. Here the difference $\bar{g}_1-g_1$ vanishes on a finite interval of $\tau _d/\tau _p$, which means that ATs with and without STDP coincide.

We now estimate the stability of unidirectional coupling $(W_{12},W_{21})=(0,1)$ in the presence of STDP. Substituting $\Delta T=(2\pi -\bar{\varphi }_2)/\omega _2$ in the stability condition (11), we have:

22 $$\begin{aligned} \bar{\varphi }_2 > 2\pi (1-Q_2). \end{aligned}$$

The unidirectional coupling is stable on the boundary of AT $g_2$ obtained with fixed synaptic weights if $\bar{\varphi }_2^A >2\pi (1 -Q_2)$ or $Q_2>T_1/(2T_2)$. This inequality cannot be met due to the assumption $\tau _d/\tau _p>1$. Then the boundary of AT $\bar{g}_2$ with the winding number $w=1/1$ in the presence of STDP is obtained from the equality $\bar{\varphi }_2 = 2\pi (1-Q_2)$:

23 $$\begin{aligned} \bar{g}_2= \omega _2 \left\{ 2 \sin ^2(\pi Q_2) \left[ \cot (\pi Q_2)-\cot (\pi T_1/T_2) \right] \right\} ^{-1}. \end{aligned}$$

This boundary is shown by yellow dashed curve in Fig. 2d,e. The difference $\bar{g}_2-g_2$ between the boundaries of ATs in the presence of STDP and without STDP as a function of a ratio $\tau _d/\tau _p$ for different values of $T_2/T_1$ is shown in Fig. 2f.

### General results near resonances

The QIF neural model made it possible to obtain analytical expressions for boundaries of ATs for arbitrary values of the natural periods $T_1$ and $T_2$ of neurons. Near resonances, similar analytical expressions can be obtained for any class I neural model. Suppose that the ratio of periods can be represented as

24 $$\begin{aligned} T_2/T_1=n+\varepsilon , \end{aligned}$$

where *n* is a natural number and $\varepsilon$ is a small parameter. For winding numbers $n=2,3,\ldots$ the parameter $\varepsilon$ is negative, and for $n=1$ it is positive. We now consider the Winfree model (6) with an arbitrary positive PRC $Z_i(\varphi ) \ge 0$. The last requirement limits our consideration to class I neurons.

#### Arnold tongues with fixed synaptic weights

Let us consider the case of fixed synaptic weights $(W_{12}, W_{21})=(1,0)$, assuming that the coupling strength *g* is small. At the instants of firing of the second neuron, the phase $\varphi _1$ of the first neuron satisfies the mapping

25 $$\begin{aligned} \varphi _1^{(k+1)} = \varphi _1^{(k)} + \omega _1 T_2+ g Z_1(\varphi _1^{(k)} + \omega _1 T_2). \end{aligned}$$

The fixed points of this map are determined by the equation $\varphi _1^{(k+1)} =\varphi _1^{(k)}\equiv \bar{\varphi }_1$. This gives $\omega _1 T_2+g Z_1(\bar{\varphi }_1+\omega _1 T_2)=0$. Substituting $\omega _1 T_2=2\pi (n+\varepsilon )$ and taking into account that the phase is defined modulo $2\pi$, we obtain

26 $$\begin{aligned} 2 \pi \varepsilon +g Z_1(\bar{\varphi }_1+2 \pi \varepsilon ) = 0. \end{aligned}$$

The critical value of the coupling strength $g=g_1$, at which synchronization occurs, is determined by the local properties of the PRC near its absolute maximum. We assume that close to the maximum the PRC $Z_1(\varphi )$ can be approximated by a parabola

27 $$\begin{aligned} Z_1(\varphi ) = Z_1^{\text {max}} - \alpha _1 (\varphi - \varphi _1^{\text {max}})^2, \end{aligned}$$

where $Z_1^{\text {max}}$ is the absolute maximum of the PRC, $\varphi _1^{\text {max}}$ is the phase value corresponding to this maximum, and the positive parameter $\alpha _1=-(1/2)[d^2 Z_1(\varphi )/d \varphi ^2]_{\varphi =\varphi _1^{\text {max}}}$. Using this approximation, we find that the stable stationary solution of the map (25) is

28 $$\begin{aligned} \bar{\varphi }_1=\varphi _1^{\text {max}}-2 \pi \varepsilon +\sqrt{(2 \pi \varepsilon /g+Z_1^{\text {max}})/\alpha _1}. \end{aligned}$$

Equating the expression under the root to zero, we find the boundaries of ATs with the winding numbers $w=n/1 \ge 2$:

29 $$\begin{aligned} g_{1} = -\frac{2 \pi }{Z_1^{\text {max}}} \varepsilon . \end{aligned}$$

The stationary phase on these boundaries is $\bar{\varphi }_1^A \approx \varphi _1^{\text {max}}$. The validity of the approximation (29) is easy to verify for the already analyzed QIF neurons. The PRC of QIF neurons is determined by the Eq. (7), so $Z_1^{\text {max}}=4/\omega _1$, and the approximation (29) gives $g_1=-(\omega _1/2) \pi \varepsilon$. The same result is obtained from the exact Eq. (14) by substituting $T_2/T_1=n+\varepsilon$ into Eq. (14) and expanding it in the small parameter $\varepsilon$.

We now consider synchronization for the unidirectional coupling with fixed synaptic weights $(W_{12}, W_{21})=(0,1)$. Here we use Eq. (24) with $n=1$ and $\varepsilon >0$. At the instants of firing of the first neuron, the phase $\varphi _2$ of the second neuron satisfies the mapping

30 $$\begin{aligned} \varphi _2^{(k+1)} = \varphi _2^{(k)} + \omega _2 T_1+ g Z_2(\varphi _2^{(k)} + \omega _2 T_1). \end{aligned}$$

The fixed points of this map are determined by the equation $\varphi _2^{(k+1)} =\varphi _2^{(k)}\equiv \bar{\varphi }_2$. This gives $\omega _2 T_1+g Z_2(\bar{\varphi }_2+\omega _2 T_1)=0$. Substituting $\omega _2 T_1=2\pi /(1+\varepsilon ) \approx 2\pi (1-\varepsilon )$ and taking into account that the phase is defined modulo $2\pi$, we obtain

31 $$\begin{aligned} -2 \pi \varepsilon +g Z_2(\bar{\varphi }_2-2 \pi \varepsilon ) = 0. \end{aligned}$$

Approximating the PRC $Z_2(\varphi )$ close to the maximum by a parabola

32 $$\begin{aligned} Z_2(\varphi ) = Z_2^{\text {max}} - \alpha _2(\varphi - \varphi _2^{\text {max}})^2, \end{aligned}$$

we find that the stable stationary solution of the map (30) is

33 $$\begin{aligned} \bar{\varphi }_2=\varphi _2^{\text {max}}+2 \pi \varepsilon +\sqrt{(-2 \pi \varepsilon /g+Z_2^{\text {max}})/\alpha _2}, \end{aligned}$$

and the boundary of AT is

34 $$\begin{aligned} g_{2} = \frac{2 \pi }{Z_2^{\text {max}}} \varepsilon . \end{aligned}$$

The stationary phase on this boundary is $\bar{\varphi }_2^A \approx \varphi _2^{\text {max}}$. For QIF neurons, $Z_2^{\text {max}}=4/\omega _2$, and the approximation (34) gives $g_2=\omega _2 \pi \varepsilon /2$. The exact Eq. (18) leads to the same result when $T_1/T_2 = 1+\varepsilon$ and $\varepsilon$ is a small parameter.

#### Arnold tongues in the presence of STDP

For the unidirectional coupling with fixed synaptic weights $(W_{12}, W_{21})=(1,0)$, the boundaries of ATs near resonances are determined by Eq. (29). In the presence of STDP, we are looking for the boundaries of ATs in the form

35 $$\begin{aligned} \bar{g}_{1} = -\frac{2 \pi }{Z_1^{\text {max}}} (1+\beta _1) \varepsilon , \end{aligned}$$

where $\beta _1$ is a small nonnegative parameter ($0\le \beta _1 \ll 1$) that needs to be determined. Note that STDP stability condition (20) is valid for the Winfree model with an arbitrary PRC, provided that the phase of the neuron is calibrated so that it is equal to $2\pi$ at the maximum of the membrane potential, i.e. the neuron fires when its phase reaches $2\pi$. In what follows, we will assume that this requirement is met. The boundaries of ATs in the presence of STDP remain unchanged ($\beta _1=0$) if the inequality $\bar{\varphi }_1^A\ge 2\pi (1-Q_1)$ is met. Substituting $\bar{\varphi }_1^A \approx \varphi _1^{\text {max}}$, and computing $Q_1$ at $T_2/T_1 \approx n$ this inequality can be written as $B_1 \le 0$, where

36 $$\begin{aligned} B_1=2\pi -\varphi _1^{\text {max}} - \frac{2\pi }{1+\tau _d/\tau _p} \left\{ 1+\frac{\tau _d}{T_1} \ln \left[ \sum _{j=0}^{n-1} \exp \left( -\frac{jT_1}{\tau _p} \right) \right] \right\} . \end{aligned}$$

For $B_1>0$, the parameter $\beta _1$ is obtained from Eqs. (28) and (35) using $\bar{\varphi }_1=2\pi (1-Q_1)$ and $g=\bar{g}_1$. Generalizing the above two cases, we get:

37 $$\begin{aligned} \beta _1 = {\left\{ \begin{array}{ll} 0 &{} \text {if } B_1 \le 0, \\ B_1^2 \alpha _1/Z_1^{\text {max}} &{} \text {if } B_1 > 0. \end{array}\right. } \end{aligned}$$

We verified the validity of the approximate Eqs. (35), (36) and (37) for QIF neurons using the exact Eq. (21). To this end, we substituted the parameters $Z_1^{\text {max}}=4/\omega _1$, $\alpha _1=1/\omega _1$ and $\varphi _1^{\text {max}}=\pi$ into the approximate equations. On the other hand, we substituted $T_2/T_1=n+\varepsilon$ into the exact Eq. (21) and, expanding it in terms of the small parameter $\varepsilon$ with the assumption $\beta _1<<1$, we obtained the same result as from the approximate equations.

Near the resonance $T_2/T_1 \approx 1$, we are looking for the boundary of AT in the presence of STDP in the form

38 $$\begin{aligned} \bar{g}_{2} = \frac{2 \pi }{Z_2^{\text {max}}} (1+\beta _2) \varepsilon . \end{aligned}$$

The value of the parameter $\beta _2$ is obtained in a similar way as described above:

39 $$\begin{aligned} \beta _2 = {\left\{ \begin{array}{ll} 0 &{} \text {if } B_2 \le 0, \\ B_2^2 \alpha _2/Z_2^{\text {max}} &{} \text {if } B_2 > 0, \end{array}\right. } \end{aligned}$$

where

40 $$\begin{aligned} B_2=2\pi /(1+\tau _p/\tau _d)-\varphi _2^{\text {max}}. \end{aligned}$$

Again, we made sure that for QIF neurons these approximate equations give the same result as the exact Eq. (23) when $T_2/T_1$ is close to one.

### Biophysical neuron models

#### Wang–Buzsáki neurons

The dynamics of neuron’s membrane potentials $v_i$ and ions activation/inactivation variables are governed by the equations^16,17^

41a $$\begin{aligned} C_{\textrm{m}} {\dot{v}}_i= & {} -g_K n_i^4 (v_i-v_{\text{K}}) - g_{\text{Na}} m_{\infty}^{3}(v_i) h_i (v_i-v_{\text{Na}}) -g_{\text{L}} (v_i-v_{\text{L}}) + I_i + g \sum _{j=1} ^2 W_{ij} S_j(t), \end{aligned}$$

41b $$\begin{aligned} \dot{h}_i= & {} \phi \left[ \alpha _h(v_i) (1-h_i) -\beta _h(v_i) h_i \right] , \end{aligned}$$

41c $$\begin{aligned} \dot{n}_i= & {} \phi \left[ \alpha _n(v_i) (1-n_i) -\beta _n(v_i) n_i \right] , \quad i=1,2 \end{aligned}$$

with the following functions

42a $$\begin{aligned} m_\infty (v)= & {} \alpha _m(v)/\left[ \alpha _m (v)+\beta _m (v)\right] , \end{aligned}$$

42b $$\begin{aligned} \alpha _\text {m} (v)= & {} 0.1 (v+35)/\left\{ 1-0.1\exp [-(v+35)]\right\} , \end{aligned}$$

42c $$\begin{aligned} \beta _\text {m} (v)= & {} 4 \exp \left[ -(v+60)/18 \right] , \end{aligned}$$

42d $$\begin{aligned} \alpha _\text {h}(v)= & {} 0.07\exp \left[ -(v+58)/20 \right] , \end{aligned}$$

42e $$\begin{aligned} \beta _\text {h}(v)= & {} 1/\left\{ 1+\exp [-0.1 (v+28)] \right\} , \end{aligned}$$

42f $$\begin{aligned} \alpha _\text {n}(v)= & {} 0.01(v+34)/\left\{ 1-\exp [-0.1(v+34)]\right\} , \end{aligned}$$

42g $$\begin{aligned} \beta _\text {n}(v)= & {} 0.125 \exp [-(v+44)/80]. \end{aligned}$$

**Figure 6:**
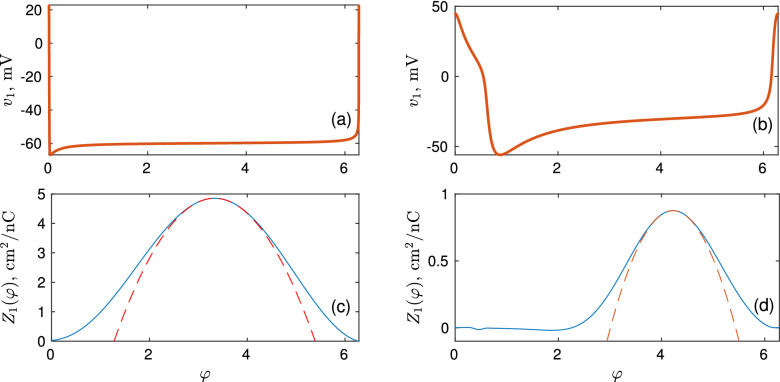
Membrane potentials and phase response curves for WB and ML neurons. The left column shows the dependence of (**a**) the membrane potential and (**c**) PRC on the phase for a periodically spiking Wang–Buzsáki neuron. Similarly, the right column shows (**b**) the membrane potential and (**d**) PRC for Morris–Lecar neuron. The red dashed curves in (**c**,**d**) show parabolic approximations of the PRCs near their maxima.

We used the parameters: $(g_{\text {K}}, g_{\text {Na}}, g_{\text {L}})= (9,35,0.1)$ $\text {mS/cm}^2$, $(v_{\text {K}},v_{\text {Na}},v_{\text {L}})=(-90,55,-65)$ mV and $\phi =5$. At direct current $I_i=I_{\text {SNIC}}\approx 0.1601$ $\upmu \text {A/cm}^2$ SNIC bifurcation occurs in an isolated ($g=0$) neuron. By increasing the current just above $I_{\text {SNIC}}$, one can effectively change the period of neural bursts in a wide range of values. For the first neuron, we chose $I_1 = 0.162677$ $\upmu \text {A/cm}^2$, which corresponds to a period of $T_1 = 500$ ms. The membrane potential and PRC for one oscillation period are shown in Fig. 6a,c, respectively. The neuron phase $\varphi$ is taken equal to zero at the maximum of the membrane potential. For almost the entire period of oscillation, the potential of the neuron is about $-60$ mV, and a sharp pulse is generated only in a narrow time interval. Since the neuron is close to the SNIC bifurcation its PRC resembles the PRC of the QIF neuron given by Eq. (7). The red dashed curve in Fig. 6c shows the parabolic fit of the PRC near the maximum. The fitting parameters in the parabolic approximation Eq. (27) are as follows: the maximum value of the PRC is $Z_1^{\text {max}} \approx 4.85$ at phase $\varphi _1^{\text {max}} \approx 3.33$ and $\alpha _1 \approx 1.15$.

#### Morris–Lecar neurons

Morris–Lecar neurons are described by the following equations^18,19^

43a $$\begin{aligned} C_{\textrm{m}} \dot{v}_i= & {} \eta_i\left[ -g_{\text {Ca}}m_{\infty }\left( v_i\right) \left( v_i-v_{\text {Ca}}\right) -g_{\text {K}}n_{i}\left( v_i-v_{\text {K}}\right) -g_{\text {L}}\left( v_i-v_{\text {L}}\right) +I_{0}\right] + g \sum _{j=1} ^2 W_{ij} S_j(t), \end{aligned}$$

43b $$\begin{aligned} \dot{n}_{i}= & {} \eta _i\frac{\phi \left[ n_{\infty }\left( v_i\right) -n_{i}\right] }{\tau _{n}}, \quad i=1,2. \end{aligned}$$

Here $v_i$ is the membrane potential, and $n_i$ is the activation variable of the *i*th neuron. The functions included in the equations are as follows:

44a $$\begin{aligned} m_{\infty }(v)= & {} 0.5\left( 1+\tanh \left[ (v-\tilde{v}_{1})/\tilde{v}_{2}\right] \right) , \end{aligned}$$

44b $$\begin{aligned} n_{\infty }(v)= & {} 0.5\left( 1+\tanh \left[ (v-\tilde{v}_{3})/\tilde{v}_{4}\right] \right) , \end{aligned}$$

44c $$\begin{aligned} \tau _{n}(v)= & {} 1/ \cosh \left[ (v-\tilde{v}_{3})/(2\tilde{v}_{4})\right] . \end{aligned}$$

We used the standard values of the parameters: $(g_{\text {Ca}}, g_{\text {K}}, g_{\text {L}})= (4,8,2)$ $\text {mS/cm}^2$, $(v_{\text {Ca}},v_{\text {K}},v_{\text {L}})=(120,-80,-60)$ mV, $(\tilde{v}_{1}, \tilde{v}_{2}, \tilde{v}_{3},\tilde{v}_{4})=(-1.2,18,12,17.4)$ mV, $I_{0}=40\, \upmu \text {A/cm}^2$, $C_{\textrm{m}}=5 \,\upmu \text {F/cm}^2$ and $\phi =1/15\,\text {ms}^{-1}$. Unlike the WB model, this model does not allow changing the firing rate of neurons in a wide range of values by changing the direct current $I_0$. To do this we introduced the time scaling parameter $\eta _i$. For the first neuron, we take $\eta _1=1$, which gives the oscillation period $T_1 \approx 86.27$ ms. For the second neuron, $\eta _2=T_1/T_2$, where $T_2$ is the desired period of the second neuron. The membrane potential and the PRC of the ML neuron are shown in Fig. 6b,d, respectively. Again, the neuron phase $\varphi$ is taken equal to zero at the maximum of the membrane potential. The red dashed curve in Fig. 6d shows the parabolic fit of the PRC near the maximum. The fitting parameters in Eq. (27) are as follows: $Z_1^{\text {max}} \approx 0.88$, $\varphi _1^{\text {max}} \approx 4.22$, and $\alpha _1 \approx 0.6$.

## Supplementary Information

Supplementary Information.

## Data Availability

All data are available on reasonable request, directed to the corresponding author, Irmantas Ratas (irmantas.ratas@ftmc.lt).

## References

[1] Schnitzler A, Gross J (2005). Normal and pathological oscillatory communication in the brain. Nat. Rev. Neurosci..

[2] Oswal A, Brown P, Litvak V (2013). Synchronized neural oscillations and the pathophysiology of Parkinson’s disease. Curr. Opin. Neurol..

[3] Stafstrom CE (2006). Epilepsy: A review of selected clinical syndromes and advances in basic science. J. Cereb. Blood Flow Metab..

[4] Jiruska P (2013). Synchronization and desynchronization in epilepsy: Controversies and hypotheses. J. Physiol..

[5] Uhlhaas PJ, Singer W (2006). Neural synchrony in brain disorders: Relevance for cognitive dysfunctions and pathophysiology. Neuron.

[6] Schultheiss NW, Prinz AA, Butera RJ (2012). Phase Response Curves in Neuroscience Theory, Experiment, and Analysis.

[7] Smeal RM, Ermentrout GB, White JA (2010). Phase-response curves and synchronized neural networks. Philos. Trans. R. Soc. B Biol. Sci..

[8] Markram H, Lübke J, Frotscher M, Sakmann B (1997). Regulation of synaptic efficacy by coincidence of postsynaptic aps and epsps. Science.

[9] Magee CJ, Johnston D (1997). A synaptically controlled, associative signal for Hebbian plasticity in hippocampal neurons. Science.

[10] Bi G, Poo M (1998). Synaptic modifications in cultured hippocampal neurons: Dependence on spike timing, synaptic strength, and postsynaptic cell type. J. Neurosci..

[11] Buonomano D, Carvalho T, Squire LR (2009). Spike-timing-dependent plasticity (STDP). Encyclopedia of Neuroscience.

[12] Morrison A, Diesmann M, Gerstner W (2008). Phenomenological models of synaptic plasticity based on spike timing. Biol. Cybern..

[13] Rinzel J, Ermentrout B (1989). Analysis of Neural Excitability and Oscillations, Chap Methods in Neuronal Modelling: From Ions to Networks.

[14] Ermentrout BG, Terman DH (2010). Mathematical Foundations of Neuroscience.

[15] Izhikevich EM (2007). Dynamical Systems in Neuroscience: The Geometry of Excitability and Bursting.

[16] Devalle F, Roxin A, Montbrió E (2017). Firing rate equations require a spike synchrony mechanism to correctly describe fast oscillations in inhibitory networks. PLoS Comput. Biol..

[17] Park Y, Ermentrout GB (2018). A multiple timescales approach to bridging spiking- and population-level dynamics. Chaos Interdiscip. J. Nonlinear Sci..

[18] Morris C, Lecar H (1981). Voltage oscillations in the barnacle giant muscle fiber. Biophys. J..

[19] Tsumoto K, Kitajima H, Yoshinaga T, Aihara K, Kawakami H (2006). Bifurcations in Morris–Lecar neuron model. Neurocomputing.

[20] Ermentrout B (1996). Type I membranes, phase resetting curves, and synchrony. Neural Comput..

[21] Ziaeemehr A, Zarei M, Sheshbolouki A (2020). Emergence of global synchronization in directed excitatory networks of type I neurons. Sci. Rep..

[22] Litwin-Kumar A, Doiron B (2014). Formation and maintenance of neuronal assemblies through synaptic plasticity. Nat. Commun..

[23] Feldman DE (2012). The spike-timing dependence of plasticity. Neuron.

[24] Rodríguez-Moreno A, Paulsen O (2008). Spike timing-dependent long-term depression requires presynaptic NMDA receptors. Nat. Neurosci..

[25] Andrade-Talavera Y, Duque-Feria P, Paulsen O, Rodríguez-Moreno A (2016). Presynaptic spike timing-dependent long-term depression in the mouse hippocampus. Cereb. Cortex.

[26] Falcón-Moya R (2020). Astrocyte-mediated switch in spike timing-dependent plasticity during hippocampal development. Nat. Commun..

[27] Zhang L, Tao H, Holt C, William H, Poo M (1998). A critical window for cooperation and competition among developing retinotectal synapses. Nature.

[28] Bi G-Q, Wang H-X (2002). Temporal asymmetry in spike timing-dependent synaptic plasticity. Physiol. Behav..

[29] Froemke RC, Dan Y (2002). Spike-timing-dependent synaptic modification induced by natural spike trains. Nature.

[30] Sjöström PJ, Turrigiano GG, Nelson SB (2001). Rate, timing, and cooperativity jointly determine cortical synaptic plasticity. Neuron.

[31] Clopath C, Büsing L, Vasilaki E, Gerstner W (2010). Connectivity reflects coding: A model of voltage-based STDP with homeostasis. Nat. Neurosci..

[32] Masuda N, Kori H (2007). Formation of feedforward networks and frequency synchrony by spike-timing-dependent plasticity. J. Comput. Neurosci..

[33] Maistrenko YL, Lysyansky B, Hauptmann C, Burylko O, Tass PA (2007). Multistability in the Kuramoto model with synaptic plasticity. Phys. Rev. E.

[34] Câteau H, Kitano K, Fukai T (2008). Interplay between a phase response curve and spike-timing-dependent plasticity leading to wireless clustering. Phys. Rev. E.

[35] Takahashi YK, Kori H, Masuda N (2009). Self-organization of feed-forward structure and entrainment in excitatory neural networks with spike-timing-dependent plasticity. Phys. Rev. E.

[36] Gilson M, Burkitt AN, Grayden DB, Thomas DA, van Hemmen JL (2009). Emergence of network structure due to spike-timing-dependent plasticity in recurrent neuronal networks. II. Input selectivity–symmetry breaking. Biol. Cybernet..

[37] Bayati M, Valizadeh A (2012). Effect of synaptic plasticity on the structure and dynamics of disordered networks of coupled neurons. Phys. Rev. E.

[38] Ratas I, Pyragas K, Tass PA (2021). Multistability in a star network of Kuramoto-type oscillators with synaptic plasticity. Sci. Rep..

[39] Popovych O, Tass P (2012). Desynchronizing electrical and sensory coordinated reset neuromodulation. Front. Hum. Neurosci..

[40] Madadi Asl M, Valizadeh A, Tass PA (2018). Delay-induced multistability and loop formation in neuronal networks with spike-timing-dependent plasticity. Sci. Rep..

[41] Babadi B, Abbott LF (2013). Pairwise analysis can account for network structures arising from spike-timing dependent plasticity. PLoS Comput. Biol..

[42] Lücken L, Popovych OV, Tass PA, Yanchuk S (2016). Noise-enhanced coupling between two oscillators with long-term plasticity. Phys. Rev. E.

[43] Winfree AT (1967). Biological rhythms and the behaviour of populations of coupled oscillators. J. Theoret. Biol..

[44] Winfree AT (2001). The Geometry of Biological Time. Interdisciplinary Applied Mathematics.

[45] Tass PA, Majtanik M (2006). Long-term anti-kindling effects of desynchronizing brain stimulation: A theoretical study. Biol. Cybern..

[46] Kromer JA, Tass PA (2020). Long-lasting desynchronization by decoupling stimulation. Phys. Rev. Res..

[47] Kromer JA, Khaledi-Nasab A, Tass PA (2020). Impact of number of stimulation sites on long-lasting desynchronization effects of coordinated reset stimulation. Chaos Interdiscip. J. Nonlinear Sci..

[48] Khaledi-Nasab A, Kromer JA, Tass PA (2022). Long-lasting desynchronization of plastic neuronal networks by double-random coordinated reset stimulation. Front. Netw. Physiol..

[49] Tass PA (2012). Coordinated reset has sustained aftereffects in Parkinsonian monkeys. Ann. Neurol..

[50] Adamchic I (2014). Coordinated reset neuromodulation for Parkinson’s disease: Proof-of-concept study. Mov. Disord..

[51] Wang J (2016). Coordinated reset deep brain stimulation of subthalamic nucleus produces long-lasting, dose-dependent motor improvements in the 1-methyl-4-phenyl-1,2,3,6-tetrahydropyridine non-human primate model of Parkinsonism. Brain Stimul..

[52] Iaccarino HF (2016). Gamma frequency entrainment attenuates amyloid load and modifies microglia. Nature.

[53] Mondragón-Rodríguez S, Gu N, Fasano C, Peña-Ortega F, Williams S (2019). Functional connectivity between hippocampus and lateral septum is affected in very young Alzheimer’s transgenic mouse model. Neuroscience.

[54] Adaikkan C, Tsai L-H (2020). Gamma entrainment: Impact on neurocircuits, glia, and therapeutic opportunities. Trends Neurosci..

[55] Andrade-Talavera Y, Rodríguez-Moreno A (2021). Synapticplasticity and oscillations in Alzheimer’s disease: A complex picture of a multifaceted disease. Front. Mol. Neurosci..

[56] Arroyo-García LE (2021). Impaired spike-gamma coupling of area CA3 fast-spiking interneurons as the earliest functional impairment in the AppNL-G-F mouse model of Alzheimer’s disease. Mol. Psychiatry.

