# Supplementary material for: Interplay of different synchronization modes and synaptic plasticity in a system of class I neurons
Source: Sci Rep. 2022 Nov 16;12:19631. doi: 10.1038/s41598-022-24001-2 (PMC9668974; doi:10.1038/s41598-022-24001-2)
Supplement: Supplementary file 1 — Supplementary Information. [file 41598_2022_24001_MOESM1_ESM.pdf]

Interplay of different synchronization modes and synaptic plasticity in a system of class I neurons

Supplementary Information

Irmantas Ratas and Kestutis Pyragas

Center for Physical Sciences and Technology, LT-10257 Vilnius, Lithuania

Supplementary note 1: Simulation of QIF neurons in the presence of noise

Numerical simulation of QIF neurons in the presence of noise was carried out to test the robustness of ATs to external perturbations. We supplemented Eq. (4) in the main text with an additional noise term $\sigma\xi_i(t)$ describing external current sources:

$$\dot{v}_i = v_i^2 + \eta_i + g\sum_{j=1}^{N} W_{ij}(t)S_j(t) + \sigma\xi_i(t). \tag{S.1}$$

Here $\xi_i(t)$ are independent white Gaussian noises with zero mean $\langle\xi_i(t)\rangle = 0$ and covariance $\langle\xi_i(t)\xi_j(t')\rangle = \delta_{ij}\delta(t-t')$, where δ_{ij} is the Kronecker delta and $\delta(t-t')$ is the Dirac delta function. The parameter σ determines the amplitude of noise. The evolution of synaptic weights is governed by STDP rule Eq. (3). For numerical simulation, the Winfree form of the Eq. (S.1) is more convenient:

$$\dot{\varphi}_i = \omega_i + gZ_i(\varphi_i)\sum_{j=1}^{N} W_{ij}(t)S_j(t) + \sigma Z_i(\varphi_i)\xi_i(t). \tag{S.2}$$

This equation should be interpreted in Stratonovich's sense[1]. Numerical simulation was carried out using the strong Euler-Heun method with the parameter $\vartheta = 0$ (see Ref.[2] for details).

As an example, here we demonstrate the effect of noise on two coupled neurons in asymptotic mode (i). Recall that in this mode, a slower neuron enslaves a faster neuron with coupling weights $(W_{12}, W_{21}) \approx (1.0)$ and both oscillate synchronously with a period equal to the period of the slow neuron. Of course, perfect synchronization of neurons in the presence of noise is impossible, since the interspike intervals of neurons fluctuate over time. We will say that mode (i) is stable in the presence of noise if the noisy neurons have equal average periods and the coupling remains unidirectional with weights (W_{12}, W_{21}) close to the values $(1,0)$. An example of the dynamics of a stable asymptotic mode (i) in the presence of noise with $\sigma = 0.01$ is presented in Fig. S.1. The synaptic weights (W_{12}, W_{21}) shown in Figs. S.1(a) and S.1(b) fluctuate slightly around the values $(1,0)$, so that these fluctuations are unnoticeable in the figure. However, the interspike intervals $\Delta t_{1,2}^{(k)}$, shown in Fig. S.1(c) as dots, fluctuate with a much larger amplitude around the mean values (dashed lines). To compare these results with the noiseless case we refer to Fig. 1(g) in the main text.

To estimate the boundaries of ATs in the presence of noise, we analyzed the dependence of the stability of mode (i) on the coupling strength g. For each fixed value of g, we performed $\mathcal{N}$ independent experiments with noisy neurons over a sufficiently large time interval $\mathcal{T}$ compared to the characteristic time scales of the system. From these experiments, the probability that mode (i) is stable was estimated as $P_1(g) = \mathcal{N}_1(g)/\mathcal{N}$, where $\mathcal{N}_1(g)$ is the number of experiments in which the noisy neurons remain in the asymptotic mode (i). An example of the dependence $P_1(g)$ for the period ratio $T_2/T_1 = 1.8$ is shown in Fig. S.2(a). For each g marked with blue asterisks, we ran $\mathcal{N} = 60$ independent simulations over the time interval $\mathcal{T} = 1.255\cdot 10^5$. We see that the dependence $P_1(g)$ has a sigmoid shape with a sharp transition from 0 to 1. For given T_2/T_1, we can define the AT boundary in the presence of noise as the value of the coupling strength $\tilde{g}$, at which the probability $P_1(\tilde{g})$ is equal to 0.5. In Fig. S.2(b), we repeated this procedure for different values of T_2/T_1, and plotted the values of $\tilde{g}$ by red dots. For comparison, in this figure, we also reproduced the results shown in Fig. 2(a), which were obtained without noise. We see that the resonant structure of ATs is preserved in the presence of noise.

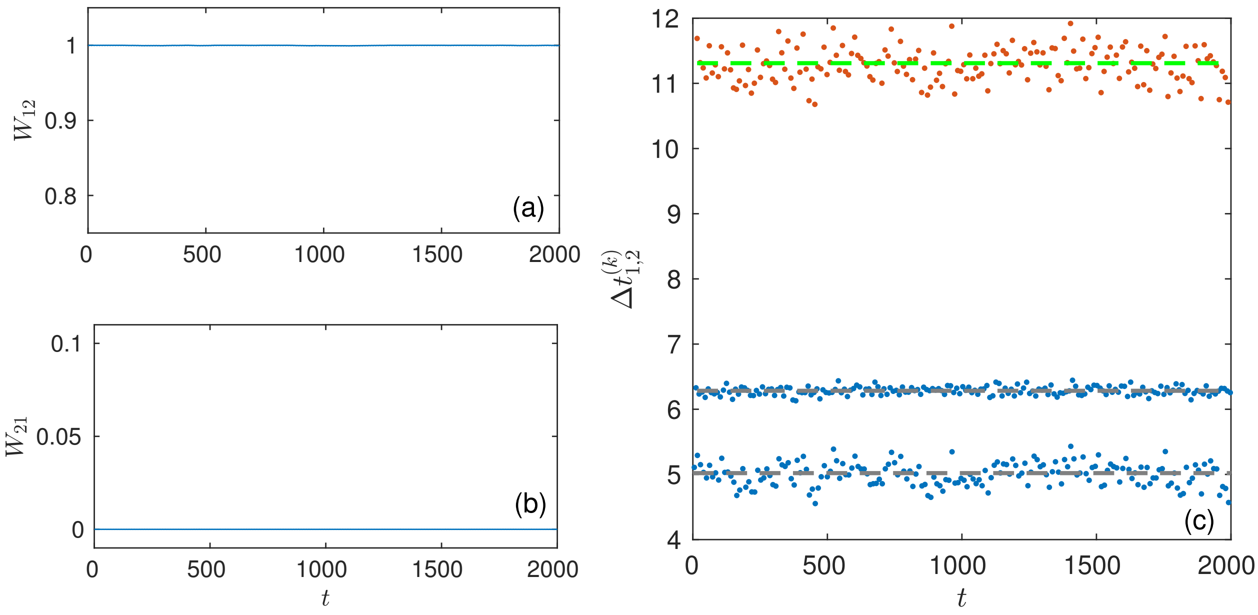

Figure S.1. Dynamics of synaptic weights (a) W_{12}, (b) W_{21}, and (c) interspike intervals $\Delta t_{1,2}^{(k)}$ of two coupled QIF neurons in the presence of noise. The blue and red dots in panel (c) show the interspike intervals of the first and second neuron, respectively. The green and grey dashed lines represent the averaged interspike values. All parameters are the same as in Fig. 1, except the ratio of the periods $T_2/T_1 = 1.8$ and the coupling strength $g = 0.55$, the noise amplitude is $\sigma = 0.01$.

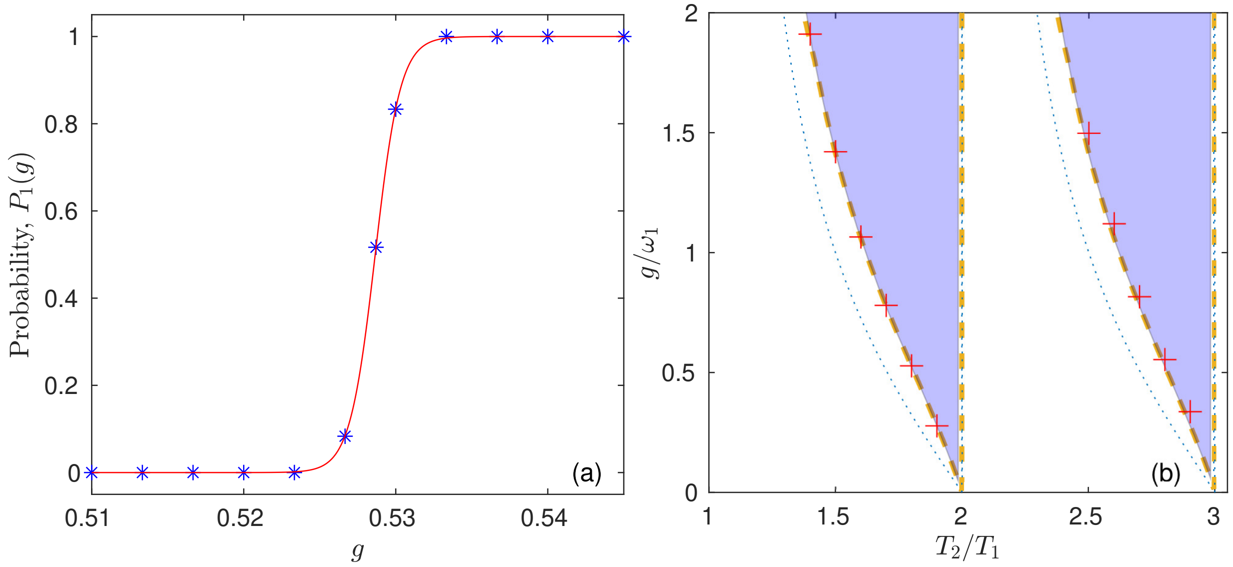

Figure S.2. (a) The probability $P_1(g)$ that mode (i) is stable in the presence of noise as a function of the coupling strength g for $T_2/T_1 = 1.8$. Blue asterisks show numerical experiments with $\mathcal{N} = 60$, $\mathcal{T} = 1.255 \cdot 10^5$, and $\sigma = 0.01$. The red curve approximates the probability with a sigmoid function. (b) Reproduction of the results from Fig. 2(a) in the main text, supplemented by red crosses, which mark the boundaries of ATs of QIF neurons with plastic synapses in the presence of noise. All parameters are the same as in Fig. S.1.

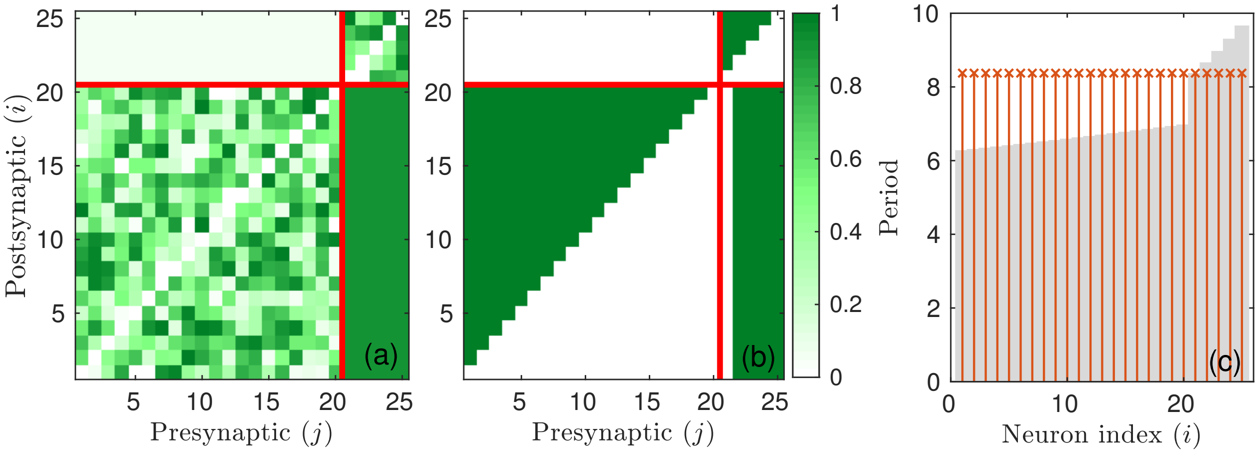

Figure S.3. An examples of a connectivity pattern emerging in a plastic network of QIF neurons with natural periods far from resonance. The natural periods of the first 20 fast neurons are equidistantly distributed in the interval $[6.28, 6.98]$, and the natural periods of the last 5 slow neurons are in the interval $[8.38, 9.67]$. (a) Initial matrix W_{ij} with partially random and partially deterministic choice of elements. (b) The asymptotic values of the matrix W_{ij} obtained from the initial matrix shown in panel (a). Panel (c) shows the distribution of natural (gray bars) and actual (vertical red lines ending in crosses) periods of neurons in the post-transient regime. The coupling strength is $g = 1.35$.

Supplementary note 2: Plastic network of QIF neurons with natural periods far from resonance and in the presence of noise

In Fig. 4 of the main text, we considered plastic networks of QIF neurons, consisting of two groups of fast and slow neurons with an approximately twofold ratio of natural periods between groups. Here we consider the same network but with a non-resonant ratio of natural periods between groups. Specifically, we assume that the natural periods of 20 fast neurons are equidistantly distributed in the interval $[6.28, 6.98]$, and the natural periods of 5 slow neurons are in the interval $[8.38, 9.67]$. The ratio of periods between the fastest neurons in these groups is 1.54, i.e. significantly different from 2. To ensure synchronization between neurons with natural periods far from resonance, larger values of the coupling strength g are required. Here we take $g = 1.35$, which is much larger than the value $g = 0.25$ used in Figs. 4. The remaining parameters are taken the same as in Fig. 4.

An example of a connectivity pattern emerging in such a network is shown in Fig. S.3. Panels (a)-(c) in this figure are similar to panels (d)-(f) in Fig. 4. The asymptotic values of the connectivity matrix W_{ij} shown in Fig. S.3(b) can be explained in terms of the asymptotic modes (i)-(iii) in the same way as it was done for the results shown in Fig. 4(e). In Fig. S.3(b) we see that the 21st neuron, the fastest in the group of slow neurons, enslaves only slower neurons. However, there are unidirectional connections from slow 22-25th neurons to fast 1-20th neurons. As a result, the entire network oscillates with the period of the slow 21st neuron [see Fig. S.3(c)]. This example shows that a slow neuron can become a pacemaker even in such a "non-resonant" network, provided that the coupling strength is large enough.

The influence of noise on the formation of the connectivity pattern for this system is shown in Fig. S.4. The simulation was carried out with the same initial matrix W_{ij} as in Fig. S.3(a). The top row corresponds to the simulation with noise amplitude $\sigma = 0.01$. We found that the asymptotic state is practically indistinguishable from the noiseless case [cf. Figs. S.4(a) and S.4(b) with Figs. S.3(b) and S.3(c)]. The middle and bottom rows show the results of simulation at a doubled noise amplitude $\sigma = 0.02$. Now the connectivity pattern starts to fluctuate in time. The middle and bottom rows show snapshots of the pattern after two rather long periods of time $t = 6.3 \cdot 10^5$ and $t = 18.8 \cdot 10^5$, respectively. Fluctuations in synaptic weights are noticeable only near the diagonal, where the natural periods of neurons are close to each other. The rest of the connections remain the same as in the noiseless case. The distribution of the average periods of neurons, shown in Fig. S.4(d) and S.4(e), also remains almost the same as in the case of no noise. Thus, even with a sufficiently large noise, the slow neuron remains the pacemaker.

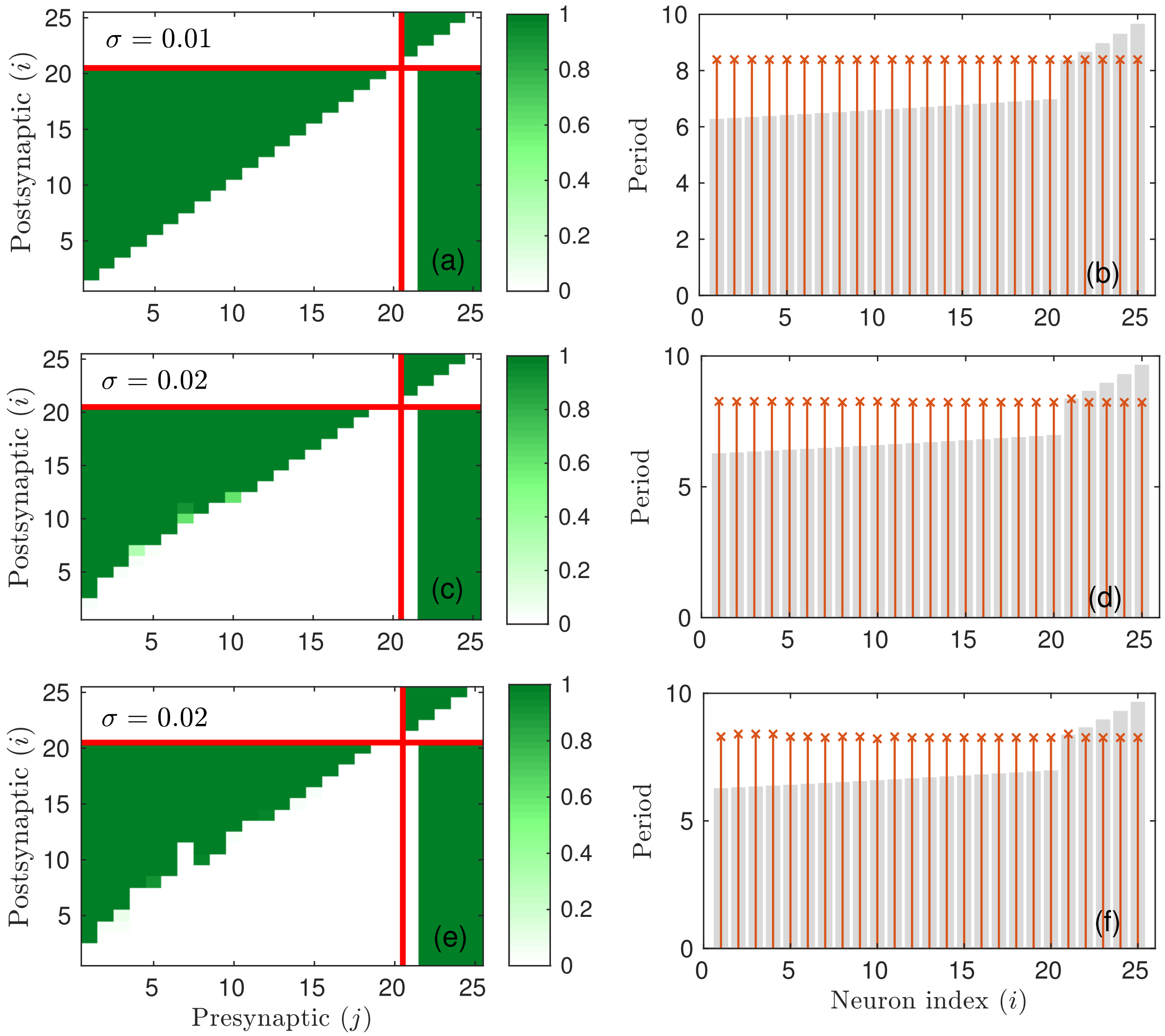

Figure S.4. Connectivity patterns in the plastic network of QIF neurons in the presence of noise. Initial conditions and all parameters are the same as in Fig. S.3. The left and right columns show, respectively, the connectivity patterns and the distribution of the average periods of neurons after a long integration time. Panels (a) and (b) show results for the noise amplitude $\sigma = 0.01$. Here the asymptotic state is practically indistinguishable from the noiseless case. Panels (c)-(f) show the results for the noise amplitude $\sigma = 0.02$. Now the connectivity pattern slowly fluctuates over time. The middle and bottom rows show snapshots of the pattern after two rather long periods of time $t = 6.3 \cdot 10^5$ and $t = 18.8 \cdot 10^5$, respectively.

References

1. Kampen, N. G. Itô versus Stratonovich. *Journal of Statistical Physics* **24**, 175–187 (1981). URL https://doi.org/10.1007/BF01007642.

2. Gilsing, H. & Shardlow, T. SDELab: A package for solving stochastic differential equations in MATLAB. *Journal of Computational and Applied Mathematics* **205**, 1002–1018 (2007). URL https://www.sciencedirect.com/science/article/pii/S0377042706004195.
